# Ketocarotenoid Production in Soybean Seeds through Metabolic Engineering

**DOI:** 10.1371/journal.pone.0138196

**Published:** 2015-09-16

**Authors:** Emily C. Pierce, Peter R. LaFayette, María A. Ortega, Blake L. Joyce, Dean A. Kopsell, Wayne A. Parrott

**Affiliations:** 1 Center for Applied Genetic Technologies and the Institute of Plant Breeding, Genetics, and Genomics, The University of Georgia, Athens, Georgia, United States of America; 2 The School of Plant Sciences, BIO5 Institute, University of Arizona, Tucson, Arizona, United States of America; 3 Plant Sciences Department, The University of Tennessee, Knoxville, Tennessee, United States of America; Instituto de Biología Molecular y Celular de Plantas (IBMCP), SPAIN

## Abstract

The pink or red ketocarotenoids, canthaxanthin and astaxanthin, are used as feed additives in the poultry and aquaculture industries as a source of egg yolk and flesh pigmentation, as farmed animals do not have access to the carotenoid sources of their wild counterparts. Because soybean is already an important component in animal feed, production of these carotenoids in soybean could be a cost-effective means of delivery. In order to characterize the ability of soybean seed to produce carotenoids, soybean cv. Jack was transformed with the *crtB* gene from *Pantoea ananatis*, which codes for phytoene synthase, an enzyme which catalyzes the first committed step in the carotenoid pathway. The *crtB* gene was engineered together in combinations with ketolase genes (*crtW* from *Brevundimonas* sp. strain SD212 and *bkt1* from *Haematococcus pluvialis*) to produce ketocarotenoids; all genes were placed under the control of seed-specific promoters. HPLC results showed that canthaxanthin is present in the transgenic seeds at levels up to 52 μg/g dry weight. Transgenic seeds also accumulated other compounds in the carotenoid pathway, such as astaxanthin, lutein, β-carotene, phytoene, α-carotene, lycopene, and β-cryptoxanthin, whereas lutein was the only one of these detected in non-transgenic seeds. The accumulation of astaxanthin, which requires a β-carotene hydroxylase in addition to a β-carotene ketolase, in the transgenic seeds suggests that an endogenous soybean enzyme is able to work in combination with the ketolase transgene. Soybean seeds that accumulate ketocarotenoids could potentially be used in animal feed to reduce or eliminate the need for the costly addition of these compounds.

## Introduction

Carotenoids are a group of naturally occurring pigments that provide distinctive red, yellow, and orange colorations to many organisms. These pigments are produced by photosynthetic algae and higher plants, as well as by non-photosynthetic bacteria, fungi, and yeast. Because of their dramatic colors and potential health benefits, carotenoids are frequently used in food products, cosmetics, and pharmaceuticals [1]. Carotenoids play a number of beneficial roles in animal health. They have strong antioxidant activity and are thought to be involved in cancer prevention [2]. Lutein and zeaxanthin carotenoids are selectively deposited in the eye as protective macular pigment, and increased dietary intake of foods rich in these carotenoids can decrease the risk of age-related macular degeneration [3]. The orange carotenoid, β-carotene, is important for its role as a precursor to vitamin A. Vitamin A deficiency leads to blindness and to increased childhood mortality rates caused by measles and chronic diarrhea. Vitamin A also plays roles in immunity, tissue development, iron metabolism, and prevention of xerophthalmia [4]. The colorless carotenoid phytoene also has nutritional value. Increased intake of phytoene can lead to a decrease in inflammatory response to UV light; moreover, mammalian cells transformed to accumulate phytoene were also shown to be less vulnerable to oxidative stress [2, 5]. It has been suggested that β-cryptoxanthin, which also has provitamin A activity, has an anabolic effect on bone that could decrease bone loss [6].

With the exception of aphids (*Aphis spp*.), animals do not biosynthesize carotenoids, but rather obtain these pigments from the diet [7, 8]. In the poultry industry, carotenoids are important feed additives for egg yolk coloration [9, 10]. In particular, canthaxanthin, a ketocarotenoid, is valued for yolk coloration. Egg yolk coloration is an important quality trait and is one of the main traits that consumers assess when determining egg quality. Consumer preference varies among cultures, with the general European trend being for a preference for darker colored yolks [11]. Three main carotenoids (lutein, zeaxanthin, and apo-carotenoic ester) are used to establish a yellow base that is then supplemented with red carotenoids (canthaxanthin, citranaxanthin, and capsanthin) to give the yolk a golden to reddish color. Canthaxanthin, which is a reddish-orange color, has much higher rates of deposition efficiency (percent of dietary intake deposited in the yolk) than the other red carotenoids, and also creates a more homogenous yolk color [12]. In chicken (*Gallus gallus domesticus*), canthaxanthin can improve fertility of breeders and reduce embryo mortality [13].

Because of the cost associated with chemical synthesis of ketocarotenoids, there has been strong interest in developing an alternate system of production. There has been some research into the use of astaxanthin-producing algae, such as *Haematococcus pluvialis*, and engineered microorganisms, such as *Escherichia coli* and *Saccharomyces cerevisiae*, to accumulate certain carotenoids, but there is still uncertainty over whether these systems can be cost-effective [14, 15, 16]. Genetically engineering plants to produce and accumulate specific carotenoids is a possible alternative. The use of soybean (*Glycine max*), which is already a component of poultry feed, could be the more cost-effective way to produce ketocarotenoids for animal agriculture. More than 90% of the annual soybean crop of the United States is used for animal agriculture. In 2011, about 50% of the soybean meal use by livestock was used for poultry feed (http://www.soystats.com; http://unitedsoybean.org). Since soybean is already a main feed component in egg production, engineering soybean to accumulate ketocarotenoids may be a viable alternative to costly chemical synthesis.

Attempts to manipulate carotenoid production have been made in a number of crop plants [17, 18, 19, 20, 21, 22, 23, 24, 25, 26, 27]. Visual selection based on endosperm color was recently used to select maize (*Zea mays*) lines with high lutein and zeaxanthin contents for possible use in egg yolk coloration [28]. Another strategy to increase carotenoid biosynthesis that has been suggested is the creation of a “metabolic sink” for carotenoids by generating carotenoid storage structures. Increased levels of lutein and β-carotene in potato (*Solanum tuberosum*) were achieved by transformation with the *Or* allele from orange cauliflower (*Brassica oleracea*), which is hypothesized to play a role in the differentiation of plastids into chromoplasts [29]. However, the main strategy thus far for engineering carotenoid production in plants has been to introduce one or more genes, usually bacterial or algal, which code for enzymes in the carotenoid biosynthetic pathway.

**Fig 1:**
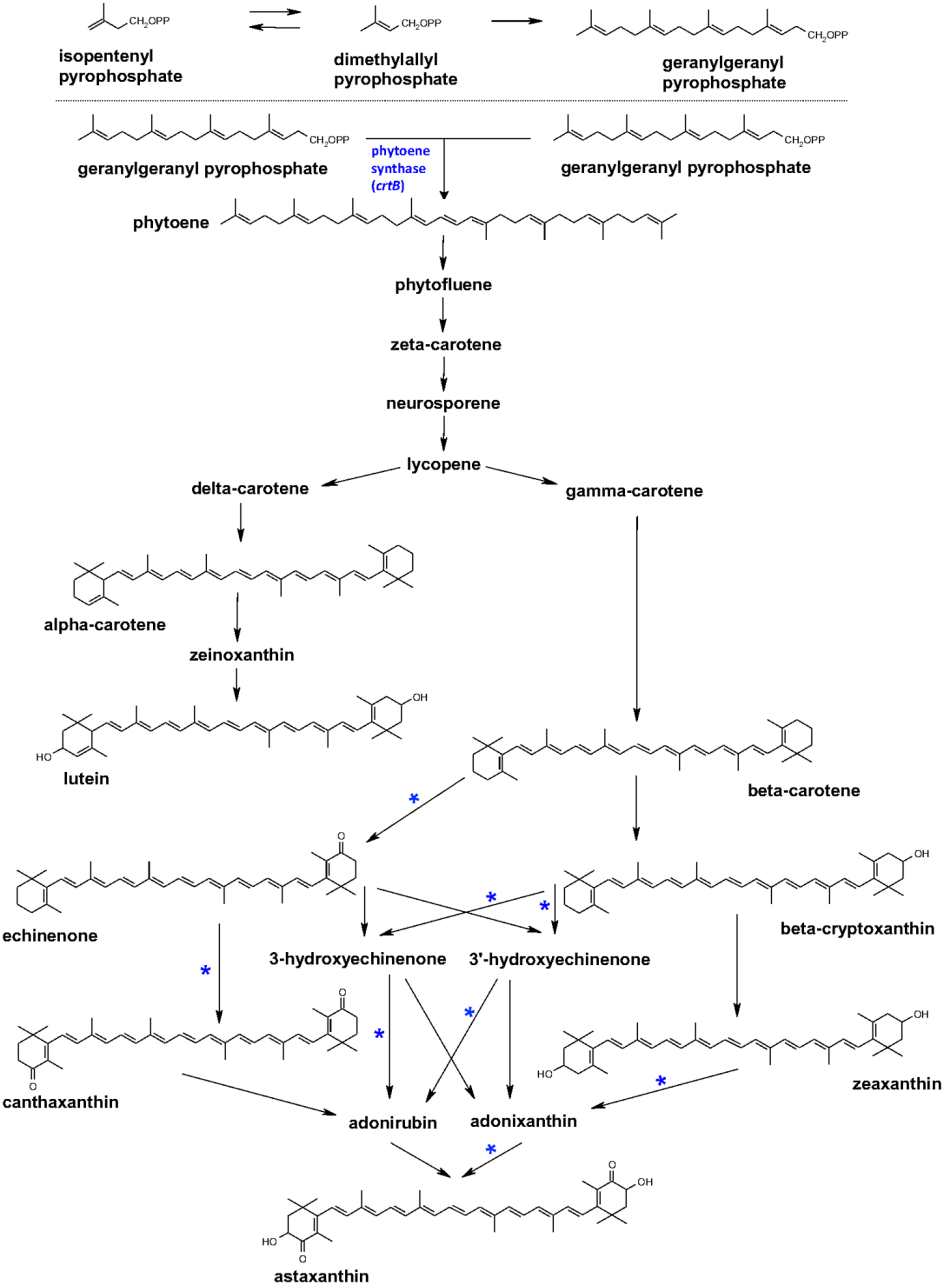
Carotenoid biosynthetic pathway. Possible pathways leading to the formation of the ketocarotenoids canthaxanthin and astaxanthin, which can be formed from β-carotene through reactions catalyzed by ketolase and hydroxylase enzymes. The *crtB* gene encodes a phytoene synthase enzyme that catalyzes the formation of phytoene. A “*” indicates a β-carotene ketolase, such as the ketolases encoded by the *crtW* or *bkt1* genes used in this study. This enzyme can use different substrates, so its final products depend on the substrates available.

Carotenoids are formed in plants through a branch of the isoprenoid biosynthesis pathway. The first committed step for carotenoid synthesis is the combination of two geranylgeranyl pyrophosphate (GGPP) molecules to form phytoene. This combination is catalyzed by phytoene synthase, known as the Psy enzyme in plants and algae and the CrtB enzyme in bacteria and fungi. Phytoene is subsequently converted into downstream carotenoids such as β-carotene, which contains two β-ring structures. Beta-carotene can be further modified to produce ketocarotenoids. Addition of a keto group to either one or both rings leads to the formation of the pigments echinenone and canthaxanthin, respectively. If both rings are both ketolated and hydroxylated, then astaxanthin is formed (Fig 1) [30]. Because the synthesis of phytoene is the first committed step in the carotenoid pathway, this step has been a common target for upregulation to shift the flow of GGPP towards carotenoid production. Transformation with *crtB*, from *Pantoea ananatis*, has been successful for the production and accumulation of β-carotene in tomato (*Solanum lycopersicum*), maize, canola (*Brassica napus*), flax (*Linum usitatissimum*), and potato [17, 18, 19, 20, 21, 22]. A phytoene synthase transgene was also used to stimulate carotenoid synthesis in rice (*Oryza sativa*) to create “Golden Rice”, which has become a widely publicized example of engineering plants to accumulate carotenoids [31, 32].

Although β-carotene is found in immature soybean seeds, changes during seed maturation lead to the degradation of β-carotene [33]. It was originally thought that all enzymes in the pathway between phytoene synthase and β-carotene were silent in the seed and therefore would need to be introduced into soybean (as was done in canola by Ravanello et al., 2003); however, the presence of β-carotene in immature soybean seeds suggests that these enzymes are expressed in developing soybean seeds [34]. Accordingly, previous work in our laboratory showed that the seed-specific expression of phytoene synthase is sufficient for β-carotene accumulation in the seeds. Soybean seeds accumulating β-carotene are described further in Schmidt et al. [35]. Enzymes that use β-carotene as a substrate are then needed for the formation of ketocarotenoids. In particular, a β-carotene ketolase is necessary for canthaxanthin production.

Several β-carotene ketolase genes are available. Members of the genus *Adonis* are the only known higher plants capable of producing ketocarotenoids, because most plants lack β-carotene ketolase [36]. Ketolase genes from *Brevundimonas* sp. strain SD212 (marine bacterium), *H*. *pluvialis* (green alga), *Paracoccus* N81106 (marine bacterium), and *Synechocystis* PCC6803 (cyanobacterium) have been used to obtain ketocarotenoids in tobacco (*Nicotiana tabacum*), carrot (*Daucus carota*), potato, canola, tomato, and maize [23,24,25,26,27]. However, most attempts have resulted in relatively low yields that are not commercially viable.

The goal of this study was to genetically engineer soybean with key enzymes in the carotenoid biosynthetic pathway to produce seeds that contain canthaxanthin at commercially viable levels. This strategy was successful in the production of seeds that accumulate ketocarotenoids as well as other compounds in the carotenoid pathway.

## Materials and Methods

**Fig 2:**
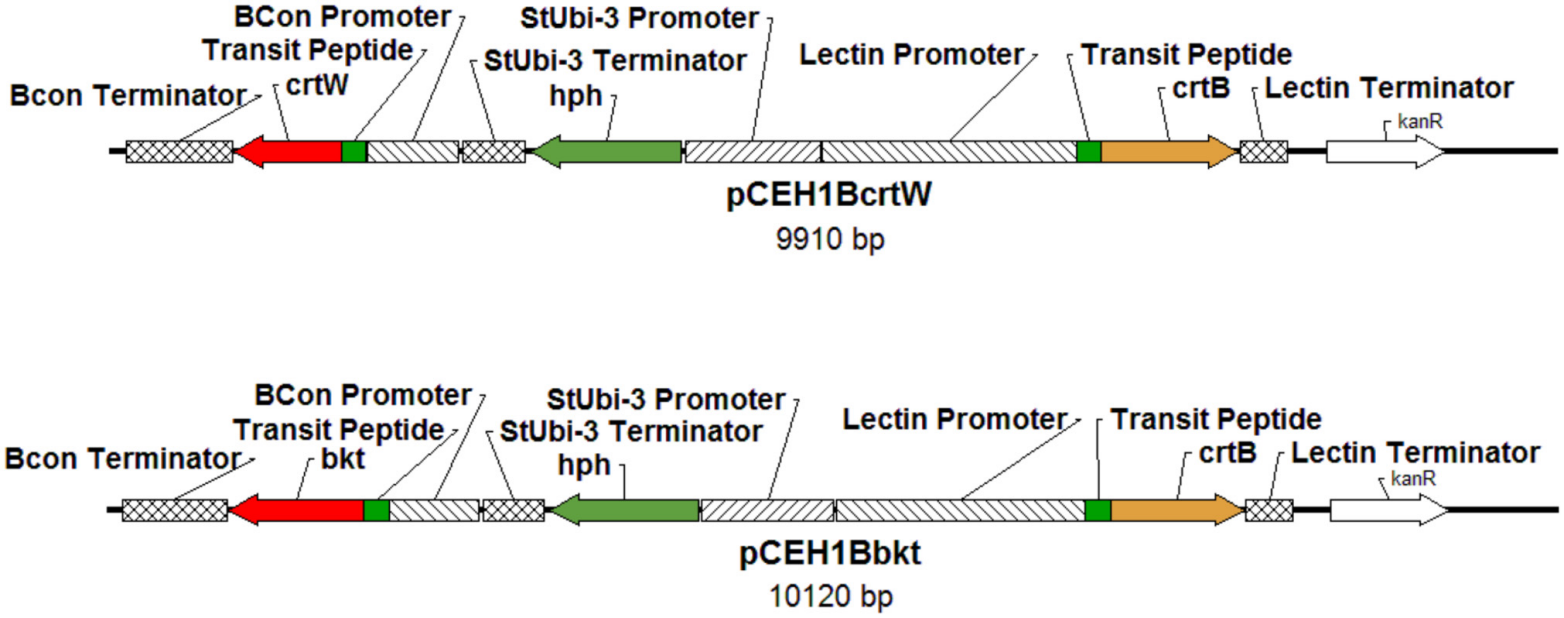
Schematic representations of transformation constructs. See *Plasmid Construction* for construct details. Abbreviations used: hph, hygromycin resistance gene; Bcon, β-conglycinin; StUbi, *Solanum tuberosum* ubiquitin; kanR, kanamycin resistance.

The carotenoid content of soybean seeds was manipulated using biolistic transformation with genes encoding phytoene synthase and ketolase enzymes under the control of lectin and β-conglycinin seed-specific promoters, respectively. To increase flow into the carotenoid pathway, the *crtB* gene of *P*. *ananatis* was used. Transformations were done with two constructs: one containing an *H*. *pluvialis* ketolase (*bkt1* (formerly *crtO*)) sequence and one containing a *Brevundimonas* sp. strain SD212 ketolase (*crtW*) sequence (Fig 2). The transit peptide for the pea (*Pisum sativum*) ribulose 1,5-biphosphate carboxylase small subunit was used to target the phytoene synthase and ketolase enzymes to the plastid [37].

### Plasmid Construction

Two genes are necessary for ketocarotenoid formation. First, phytoene synthase is needed to obtain β-carotene, and a β-carotene ketolase is needed to produce canthaxanthin. The *Brevundimonas* sp. strain SD212 *crtW* coding sequence was synthesized from GenBank (AB181388.1). GeneArt (Invitrogen) codon-optimized this sequence for expression in soybean (KC954630). To obtain the *bkt1* coding sequence, RNA was extracted from astaxanthin-producing *H*. *pluvialis* using the Spectrum Plant Total RNA Kit (Sigma-Aldrich). This RNA was then used as the template for the synthesis of cDNA by SuperScript III Reverse Transcriptase (Invitrogen) using an oligo-dT primer. Polymerase chain reaction was used to amplify the cDNA from *bkt1* (X86782.1) using gene-specific primers (5’-GTTTGTGCGCCTCGAC-3’/5’CCAGCTAGGCAGGAACCA-3’). The *bkt1* and *crtW* coding sequences were fused to the pea ribulose 1,5-biphosphate carboxylase small subunit transit peptide [37] sequence (which in the case of the *bkt1* construct contained a native intron) and cloned in between the soybean seed-specific β-conglycinin promoter [38] and terminator sequences in the previously constructed pBeta vector [35]. The BconP:TP:codingsequence:BconT cassettes were then each cloned into pCEH1B, a pSMART HCKan (Lucigen) based vector previously modified to contain *crtB* (*P*. *ananatis)* regulated by the soybean lectin promoter and terminator as well as a hygromycin resistance gene under control of the potato (*Solanum tuberosum*) ubiquitin promoter and terminator for use in selection of transgenic events (Fig 2) [39].

### Soybean Transformation

Soybean transformation procedures were modified from those of Trick et al. [40]. Briefly, somatic embryos were induced from translucent green immature cotyledons from zygotic embryos (≤ 5 mm) that were separated from each other by removing the end containing the embryonic axis. These cotyledons were placed flat side up on MSD40 medium. The resulting somatic embryos were transferred to MSD20 medium for proliferation and maintenance. Four days before bombardment, approx. 100 mg of small, compact, globular-stage repetitive embryos from cultures that were less than three months old were arranged in the center of a plate of MSD20 to form a disk about three centimeters in diameter. The lid was removed from the plate 20 min before shooting in a laminar flow hood to allow drying of the embryos. Each plate was then shot once at 7584 kPa (1100 psi) with approximately 50 ng of DNA precipitated on 667 μg of 0.6-μm diameter gold particles. A flight distance of six cm and Hg vacuum of 68.6 cm (27 in) was used.

After one-week, the tissue was divided equally into four 125-mL Erlenmeyer flasks containing 25 mL of FNL medium supplemented with 20 mg L^-1^ hygromycin-B [41]. Flasks were placed on a shaker at 125 rpm with a light intensity of 1–6 μE m^-2^ s^-1^, and a 23 h light-1 h dark photoperiod. The medium was replaced weekly. After six to eight weeks, green clusters were selected and transferred to individual flasks. When enough tissue was present, a sample was removed from the flask for DNA extraction using a modified CTAB method [42]. The presence of the transgenes was verified using primers (5’-CTCAATCATGCGGTCGAAAC-3’/5’-CAATGACAATCACTAGCG-3’) for *crtB*, (5’-TGACTGCTGCTGTTGCTGAAC-3’/5-’CGCTCTTTCTTTCCGGATTCGT-3’) for *crtW*, (5’-AATCAAGCTTCCGACCTCCTTGG-3’/5’-TAGGCAGGAACCAGACCT-3’) for *bkt1*. PCR conditions consisted of initial denaturation for 4 min at 94°C, followed by 35 cycles of denaturation for 30 s at 94°C, annealing for 30 s at 54°C, and extension for 1 min at 72°C, followed by a final extension step of 7 min at 72°C.

If the transgenes were present, the tissue was moved forward to undergo histodifferentiation in SHaM liquid medium for five weeks [43]. After seven days of desiccation, the embryos were moved to MS0 medium for germination. When both shoots and roots were present, they were moved to GA-7 boxes and then eventually to soil. After the plants were hardened off, they were transferred to the greenhouse for further growth and seed production [40].

### Carotenoid Extraction and HPLC Analysis

Seeds from two transformation events were sent to Craft Technologies, Inc. (http://www.crafttechnologies.com) for carotenoid HPLC analysis. The remainder of the HPLC analysis was performed by extracting pigments from soybean seeds following the procedure of Kurilich and Juvik [44] with slight modifications. Single soybean seeds were ground, and the tissue was placed in a test tube (20 x 150 mm) and re-hydrated with 6 mL EtOH stabilized with 0.1% BHT. Addition of 0.8 mL of the internal standard ethyl-β-8´-*apo*-carotenoate (Sigma) was used to determine extraction efficiency. Tubes were vortexed for 1 min before being capped and placed in a water bath at 85°C for 5 min, or until the ethanol was brought to boiling. Tubes were removed from the bath and 0.18 mL of 53% KOH was added for saponification. Tubes were vortexed for 1 min and returned to the bath for 10 min. After saponification, tubes were cooled in an ice bath for 2 min before addition of 3 mL of cold deionized water and 3 mL of hexane. Tubes were vortexed for 1 min and placed into a clinical centrifuge at 600 x *g* for 10 min. A Pasteur pipette was used to remove the partitioned hexane layer, which was transferred to a separate test tube. The addition of 3 mL of hexane to the sample tubes was made and the centrifugation step was repeated twice more. The combined hexane fractions were dried under a stream of nitrogen gas and brought up to a final volume of 5 mL with 11% methyl *tert*-butyl ether (MTBE), 88.9% MeOH, and 0.1% triethylamine (TEA). A 2-mL aliquot was filtered through a 0.2-μm Econofilter PTFE 25/20 polytetrafluoroethylene filter (Agilent Technologies) using a 5-mL syringe prior to high performance liquid chromatography (LC) analysis.

High-performance liquid chromatography separation parameters and pigment quantification followed procedures of Kopsell et al. [45]. An Agilent 1200 series LC unit with a photodiode array detector (Agilent Technologies) was used for pigment separation. The column used was a 250 x 4.6 mm i.d., 5 μm analytical scale polymeric RP-C_30_, with a 10 x 4.0 mm i.d. guard cartridge and holder (ProntoSIL, MAC-MOD Analytical Inc.), which allowed for effective separation of chemically similar carotenoid compounds. The column was maintained at 30°C using a thermostatted column compartment. All separations were achieved isocratically using a binary mobile phase of 11% methyl *tert*-butyl ethanol (MTBE), 88.9% MeOH, and 0.1% triethylamine (TEA) (v/v). The flow rate was 1.0 mL per min, with a run time of 53 min, followed by a 2 min equilibration prior to the next injection. Eluted compounds from a 10-μL injection loop were detected at 453 nm, and data were collected, recorded, and integrated using ChemStation Software (Agilent Technologies)[46]. Peak assignment for individual pigments was performed by comparing retention times and line spectra obtained from photodiode array detection using external standards (ChromaDex Inc.). The concentration of the external pigment standards were determined spectrophotometrically using methods described by Davies and Köst [47]. Slurried Spinach 2385 standard reference material (National Institute of Science and Technology,) was used for method validation.

### RNA Analysis

RNA was extracted from non-transgenic soybean cv. Jack somatic cotyledonary-stage embryos and soybean cv. Jack somatic cotyledonary embryos that had been transformed with pCEH1B+*crtW*. RNA extraction was performed using TRI Reagent solution (Sigma-Aldrich) according to the protocol in the TRI Reagent Solution Manual. cDNA synthesis was then carried out using an oligo-dT primer and the GoScript Reverse Transcription System (Promega). A BLAST search was used to compare a *H*. *pluvialis* β-carotene hydroxylase coding sequence (GenBank AF162276.1) to the nucleotide collection for soybean (http://www.ncbi.nlm.nih.gov). Primers were then designed to a predicted soybean β-carotene hydroxylase sequence (primers align to sequences of both GenBank accession number NM_001250494.1 and BT098487.1). These gene-specific primers (5’-TTCCTTGTGGCACATGCACGAG-3’/5’-TCATGAACCGGACCTGATTCTCC-3’) were used for reverse transcriptase-PCR to amplify a portion of a soybean β-carotene hydroxylase (Glyma10g37560) coding sequence using GoTaq Green Master Mix (Promega).

RT-PCR was done to verify that the transgenes were expressed in the events producing ketocarotenoids (as determined by HPLC). Tissue was not available for the crtw-2-60, bkt-4-16, and CW-1-12B events. T1 seeds were planted and total RNA was isolated from immature orange-colored cotyledons (40–90 mg fresh weight) from these events using the Quick-RNA Mini Prep kit (Zymo Research) with DNaseI digestion. The cDNA synthesis for RT-PCR was done using the GoScript Reverse Transcription System with oligo (dT) priming (Promega). Orange cotyledons were selected for this analysis except in the case of the non-transformed Jack sample. RT-PCR was done for *crtB* (primers: 5’-CTCAATCATGCGGTCGAAAC-3’/5’-CAATGACAATCACTAGCG-3’) and for either *crtW* (primers: 5’-TGACTGCTGCTGTTGCTGAAC-3’/5-’CGCTCTTTCTTTCCGGATTCGT-3’), or *bkt1* (primers: 5’-AATCAAGCTTCCGACCTCCTTGG-3’/5-’CGCTCTTTCTTTCCGGATTCGT-3’) as applicable. An RT-PCR reaction was also done with a non-transgenic cv. Jack control sample for *crtB*. Control reactions without reverse transcriptase were also done for all samples.

### Statistical Analysis of HPLC Results

Data was analyzed on IBM SPSS statistics. Lutein, phytoene, β-carotene, canthaxanthin, and α-carotene levels were compared between events from the same construct and between constructs. *T*-tests (p >0.05) were used to compare between two groups, and a one-way analysis of variance (p < 0.05) was used to evaluate mean differences when more than two groups were compared. A Duncan’s test (p < 0.05) for multiple comparisons was used to detect significant differences between pCEH1B+*crtW* events.

## Results

**Fig 3:**
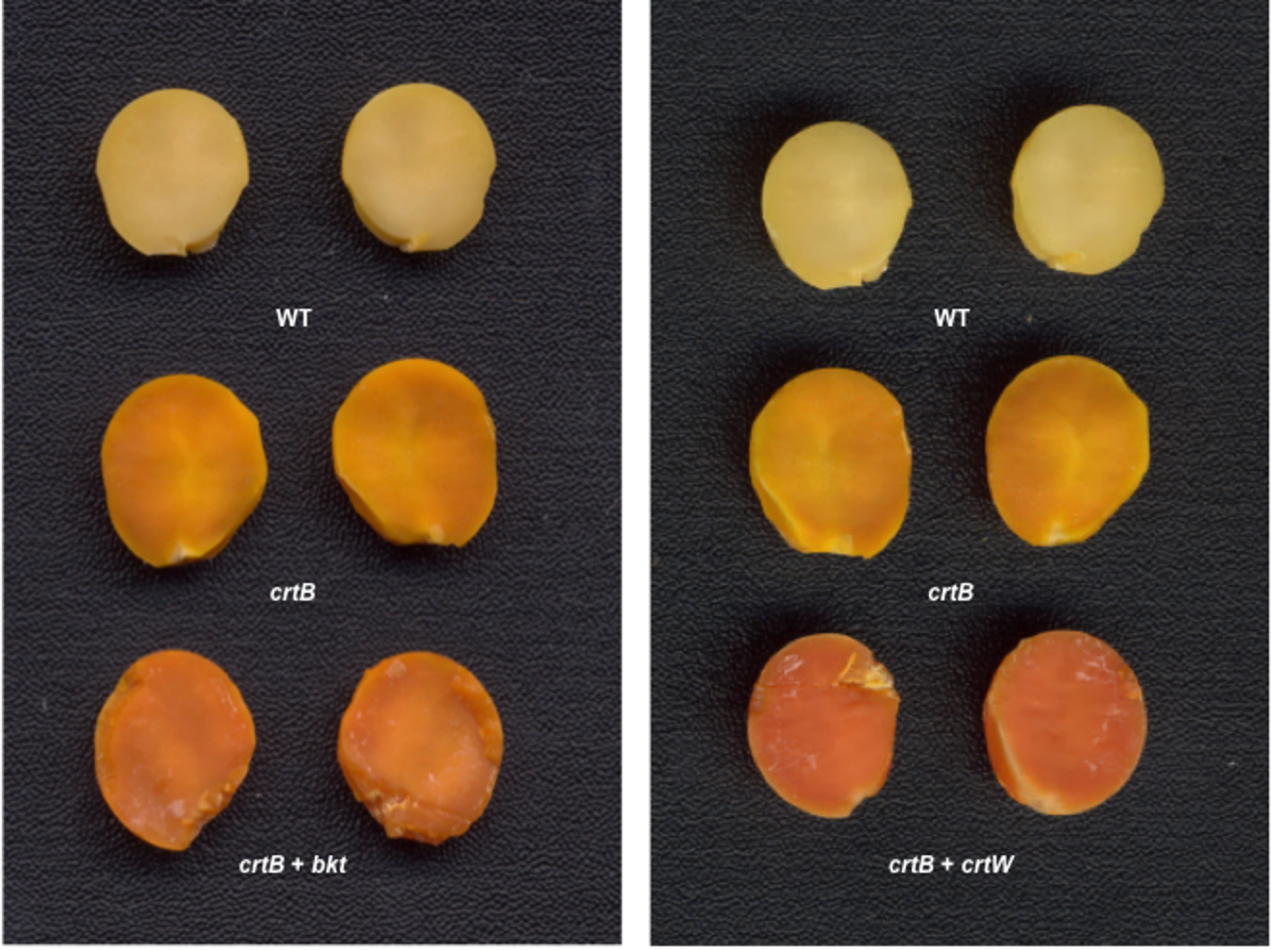
Soybean seeds from events producing canthaxanthin. The top row in each panel is of wild-type (WT) soybean cultivar Jack. The second row is of carotene-accumulating seeds resulting from transformation with *crtB*. The third row is of seeds containing canthaxanthin after transformation with pCEH1B+*bkt* (left panel) or pCEH1B+*crtW* (right panel). The cotyledons from one seed have been separated in each case.

A dramatic color change was seen in transgenic T1 seeds resulting from transformation with both constructs (Fig 3). Based on HPLC results, soybean transformation with genes encoding phytoene synthase and ketolase enzymes resulted in soybean seeds accumulating ketocarotenoids in addition to β-carotene, phytoene, β-cryptoxanthin, lutein, α-carotene, and lycopene. Of the events analyzed using HPLC, three events resulting from transformation with the *H*. *pluvialis* ketolase (*bkt1*) and seven events resulting from transformation with the *Brevundimonas* SD212 ketolase (*crtW)* accumulated canthaxanthin. Lutein was the only of these carotenoids detected by HPLC in the cv. Jack non-transgenic sample (15 μg g^-1^). Subsequent generations for one of the *Brevundimonas* SD212 ketolase events (crtw-1-32) and from one event transformed with the *H*. *pluvialis* ketolase (bkt-50) were also found to accumulate β-carotene, phytoene, lutein, α-carotene, and canthaxanthin based on HPLC.

**Fig 4:**
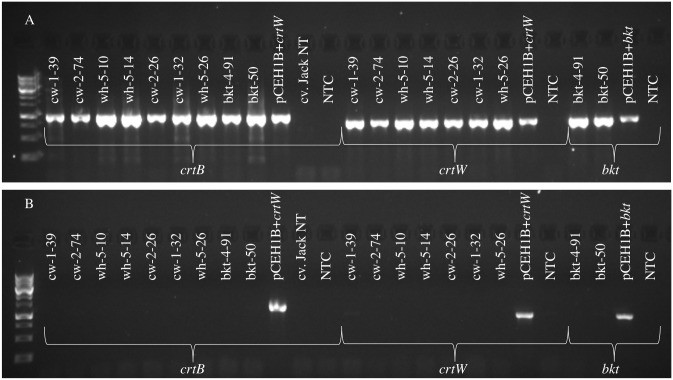
RT-PCR analysis of carotenoid biosynthetic pathway RNA from transgenic soybean seeds. Panel A shows RT-PCR products for *crtB*, *crtW*, and *bkt1*. Lower panel (B) shows reactions without reverse transcriptase. Cv. Jack NT is a non-transgenic control sample. NTC stands for no template control.

Data for carotenoids based on HPLC are shown in Tables 1–6. All of the events in this study producing ketocarotenoids that were analyzed by RT-PCR show expression of *crtB* and either *crtW* or *bkt1*, depending on the construct (Fig 4).

**Table 1:** Amounts of compounds from the carotenoid biosynthetic pathway detected by HPLC in transgenic T1 soybean seeds resulting from transformation with pCEH1B+*crtW*.

pCEH1B+*crtW* Event	lutein	phytoene	β-carotene	canthaxanthin	α-carotene
crtw-1-39	9^a^	9^a^	60^a^	4^a^	4^a^
*crtw-2-74	8	9	218	10	25
wh-5-10	4^a^	12^a^	292^bc^	24^b^	50^bc^
wh-5-14	7^a^	36^b^	741^d^	17^b^	137^cd^
crtw-2-26	6^a^	6^a^	136^b^	14^ab^	19^a^
crtw-2-60	18^a^	41^b^	594^cd^	10^a^	93^c^
*crtw-1-32	17	83	666	52	97

*this event was excluded from statistical analysis because only one seed was analyzed.

Amounts are in μg compound/g dry seed and are based on the average of two individually-analyzed seeds from the same event. Different lower case letters beside values indicate significant differences between events at the 0.05 level based on one-way ANOVA and a post-hoc Duncan’s test (p > 0.05).

**Table 2:** Amounts of compounds from the carotenoid biosynthetic pathway detected by HPLC in transgenic T1 soybean seeds resulting from transformation with pCEH1B+*bkt*.

pCEH1B+*bkt* Event	lutein	phytoene	β-carotene	canthaxanthin	α-carotene
*bkt-4-91	5	12	139	18	24
bkt-50	8^a^	44^b^	558^b^	23^a^	111^b^
bkt-4-16	8^a^	29^a^	195^a^	45^a^	47^a^

*this event was excluded from statistical analysis because only one seed was analyzed.

Amounts are in μg compound/g dry seed and are based on the average of two individually-analyzed seeds from the same event. Different lower case letters beside values indicate significant differences between the two events at the 0.05 level based on a *t*-test.

**Table 3:** Amounts of compounds from the carotenoid biosynthetic pathway detected in transgenic T1 soybean seeds resulting from transformation with pCEH1B+*crtW* based on HPLC analysis by Craft Technologies.

pCEH1B+*crtW* Event	lutein	phytoene	β-carotene	canthaxanthin	α-carotene	astaxanthin
CW-1-12B	6	181	429	5	63	7
WH-5-26B	1	39	160	7	27	2

Amounts are in μg compound/g dry seed and are based on pooled seed samples for each event. For each construct, this pool was made up of 10 grams of ground seed.

**Table 4:** Amounts of compounds from the carotenoid biosynthetic pathway detected in T2 transgenic soybean seeds from an event resulting from transformation with pCEH1B+*crtW*.

pCEH1B+*crtW* Event	Replicate	lutein	phytoene	β-carotene	canthaxanthin	α-carotene
crtw-1-32 (T2)	1	4	12	239	6	34
crtw-1-32 (T2)	2	5	12	335	6	40
crtw-1-32 (T2)	3	6	17	398	6	61
crtw-1-32 (T1)	-	17	83	666	52	97

Amounts are in μg compound/g dry seed. T1 data is based on a single seed sample. Three biological replicates were done for the T2 seed, each consisting of a pooled sample of two seeds with the exception of replicate 3, which contained only one seed. The plants producing these seeds were grown in greenhouse conditions. T1 and T2 indicate seed generation. HPLC results from the T1 generation of this event are shown for comparison.

**Table 5:** Amounts of compounds from the carotenoid biosynthetic pathway detected in T3 transgenic soybean seeds from an event resulting from transformation with pCEH1B+*bkt*.

pCEH1B+*bkt* Event	Replicate	lutein	phytoene	β-carotene	canthaxanthin	α-carotene
bkt-50 (T3)	1	4	10	310	10	49
bkt-50 (T3)	2	5	16	462	14	66
bkt-50 (T3)	3	5	12	322	11	44
bkt-50 (T1)*	-	8^a^	44^a^	558^a^	23^a^	111^a^
bkt-50 (T3)*		5^b^	13^b^	365^b^	12^b^	53^b^

Amounts are in μg compound/g dry seed. Three biological replicates were done, each consisting of a pooled sample of two seeds. The plants producing these seeds were grown in field conditions. T1 and T3 indicate seed generation. HPLC results from the T1 generation of this event are shown for comparison.

*Indicates that this data was used in *t*-test analysis. Letters beside values indicate significant differences at the 0.05 level based on a *t*-test when the two generations were compared.

**Table 6:** Amounts of compounds from the carotenoid biosynthetic pathway detected in transgenic T1 soybean seeds resulting from transformation with pCEH1B+*crtW* and pCEH1B+*bkt*.

Transgenic construct	Number of events	lutein	phytoene	β-carotene	canthaxanthin	α-carotene
pCEH1B+*crtW*	5	8 ± 6.2	21 ± 15.9	365 ± 292.9	11 ± 6.9	61 ± 57.4
pCEH1B+*bkt*	2	9 ± 2.2	39 ± 15.8	378 ± 232.1	34 ± 17.7	74 ± 35.2

Values are mean ± standard deviation. No significant differences between constructs were detected in a *t*-test (p >0.05).

**Fig 5:**
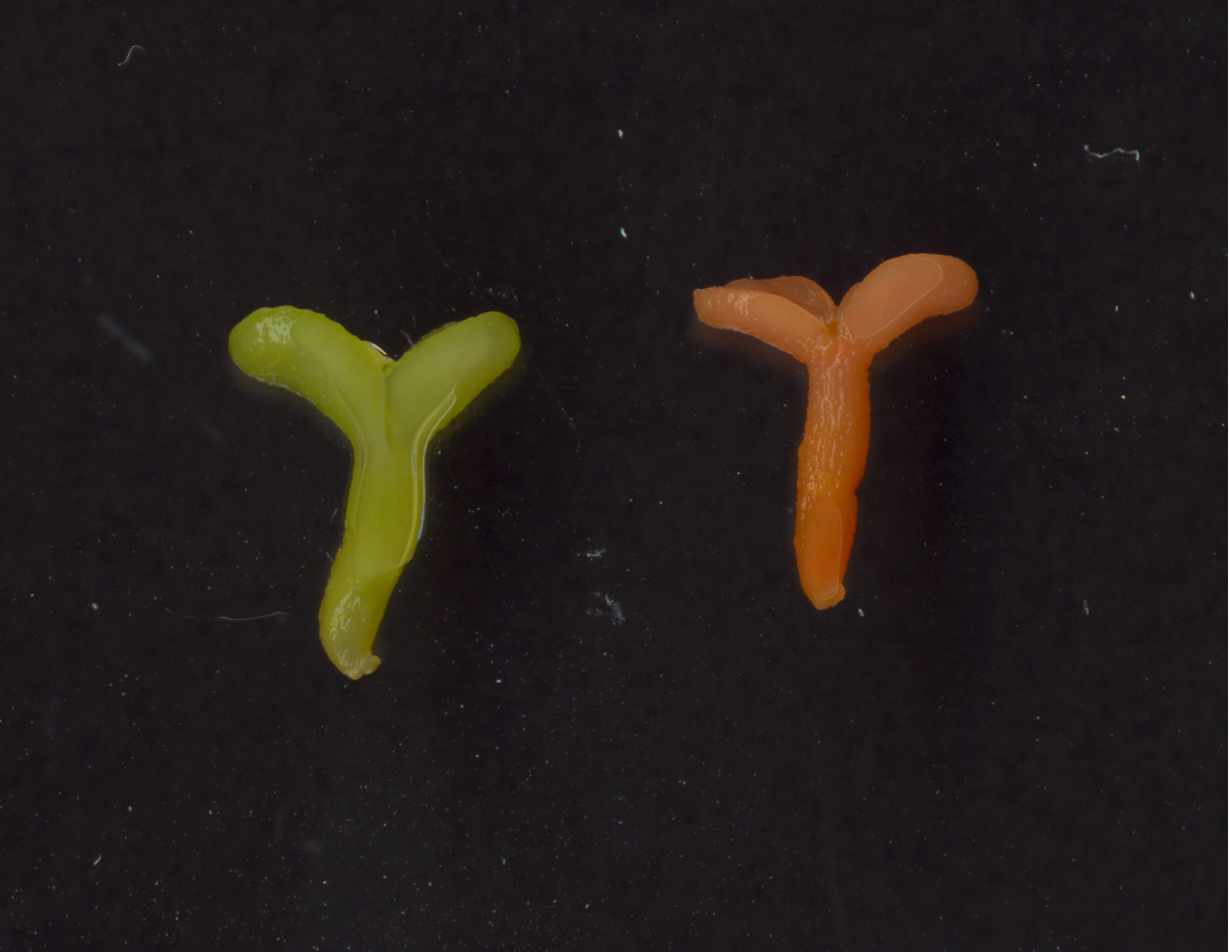
Soybean somatic embryo accumulating carotenoids. From left to right: non-transformed Jack somatic embryo, somatic embryo from Jack transformed with pCEH1B+*crtW*. The embryos in this picture were imaged approximately 5 months after bombardment.

Color change resulting from changes in carotenoid production was also seen in somatic embryos in tissue culture (Fig 5). Because soybean somatic embryos mimic seed development [43], it is not surprising that products resulting from the expression of transgenes from seed-specific promoters can be seen in these embryos. Preliminary results with thin layer chromatography of extracts from some of these colored cotyledonary-stage embryos suggested that these embryos were accumulating astaxanthin, a valuable red carotenoid, in addition to other carotenoids. The accumulation of astaxanthin was unexpected, as this requires the action of a β-carotene hydroxylase on canthaxanthin, and no hydroxylase transgene was included in the transformation constructs. Reverse-transcriptase PCR showed that a soybean β-carotene hydroxylase gene was expressed in both transgenic and non-transgenic soybean cotyledonary-stage somatic embryos. Separate analysis by Craft Technologies of two events transformed with the *crtW* construct detected canthaxanthin, astaxanthin, lutein, β-carotene, phytoene, α-carotene, lycopene, and β-cryptoxanthin in the transgenic seeds (see Table 3).

## Discussion

The *crtB* transgene encoding a phytoene synthase gene was the only transgene used in this study which encodes an enzyme that acts upstream of β-carotene production in the carotenoid biosynthetic pathway. Nevertheless, β-carotene accumulation in the analyzed dried seeds reached levels between 60–741 μg g^-1^. In contrast, Kim et al. [48] used a bicistronic recombinant gene to simultaneously express phytoene synthase from bell pepper (*Capsicum annum*) and *crtI* from *P*. *ananatis* under the control of the β-conglycinin promoter. Their highest level of β-carotene accumulation was 112 μg g^-1^, even though they used two genes for this purpose. In canola, seed-specific expression of *crtB* produced 314–949 μg g^-1^ fresh weight of β-carotene [22]. When additional transgenes coding for enzymes in the pathway between phytoene and β-carotene were used in conjunction with *crtB*, carotenoid ratios were altered compared to the use of *crtB* alone, but the overall levels remained the same and did not lead to higher levels of β-carotene [34]. Additionally, a previous study in which soybean seeds were engineered with *crtB* alone shows that *crtB* by itself leads to large increases in β-carotene in soybean seeds [35]. The accumulation of β-carotene in the transgenic seeds in this study therefore supports the findings of these two previous studies. The transgenic seeds in this study also accumulated phytoene and α-carotene, which were not detected in the non-transgenic control seeds. Similar results were seen in soybean seeds engineered with *crtB* alone; these seeds accumulated measurable amounts of phytoene and α-carotene while non-transgenic control seeds did not [35].

In contrast to the high levels of β-carotene, canthaxanthin and astaxanthin accumulated in the analyzed seeds at 5–52 μg g^-1^ and 2–7 μg g^-1^, respectively. Additionally, levels of canthaxanthin were lower in the T2 and T3 seeds tested than those found in the T1 seeds from the same events. However, it should be noted that the HPLC analysis was done on the T1 seed and then a separate HPLC analysis was done using the T2 and T3 seed. The T3 seed from the bkt-50 event were produced under field conditions, whereas all other seed in this study were produced under greenhouse conditions. Therefore it is not possible to draw conclusions about canthaxanthin production across generations based on this study.

While the plants were indistinguishable from wild type, the seeds appear to have lower germination rates at times. If so, lower germination could provide an additional challenge to ketocarotenoid production in soybean. However, it is clear that the genetic potential to achieve desirable levels of canthaxanthin production is present. Canthaxanthin is typically supplemented in the layers’ diets for yolk coloration at levels between two and eight mg kg^-1^ of diet [49]. Supplementation of 6 mg canthaxanthin per kg breeder feed can improve reproductive performance and reduce oxidative stress in chicks [13, 50]. If a six mg canthaxanthin per kg diet rate of supplementation is assumed, it is possible to calculate the amount of canthaxanthin that might be needed per g of soybean seed if these seeds were to supply all of the necessary canthaxanthin. Around 20% of layer feed is soybean meal. If 50% of the regular soybean used in feed was replaced with full-fat soybean producing canthaxanthin, approximately 60 μg canthaxanthin would be needed per g soybean seed. Thus, if the canthaxanthin levels seen in the T1 seed could be stabilized, the soybean seeds produced in this study could conceivably be used in animal feed as a source of ketocarotenoids. However, layer feeding trials will be needed to ensure that the carotenoids are bioavailable. Although canthaxanthin levels in seed from the current study appear to be high enough to supplement poultry diets, higher levels would permit the use of lower amounts of carotenoid-containing soybean meal in the feed rations. The inclusion of further strategies, such as a strategy for increasing carotenoid storage structure formation in the seed or the use of alternate ketolases, may help increase the accumulation of ketocarotenoids.

Finally, one unexpected result was the presence of astaxanthin in the soybean seeds, given there was no hydroxylase transgene. Astaxanthin was only detected in samples analyzed by Craft Technologies, but not in our analysis, possibly because the methods used in our analysis resulted in the coelution of astaxanthin and lutein. Differences in extraction methods will impact release of lipid-soluble carotenoid compounds from the seed matrix and also stability of the compounds in the extraction solvent. Differences in HPLC procedures (equipment parameters, mobile phase eluents, and/or detection wavelengths) will also influence results among labs. Results for carotenoid compounds differed among the labs used in the study, but still demonstrated the impacts from the transgenic events in the soybean seeds. This appearance of astaxanthin in our transgenic embryo and seed samples suggested that an endogenous soybean β-carotene hydroxylase, which could potentially be encoded by Glyma10g37560, was functioning in the seed. These results indicate that astaxanthin can be produced in soybean, with higher levels perhaps achieved through the overexpression of Glyma10g37560 or the use of additional hydroxylase transgenes. Astaxanthin is important for the aquaculture industry [51], and astaxanthin-producing soybean could become an efficient source of this carotenoid, permitting aquaculture to depend more on a land-based diet. Collectively, these results show that metabolic engineering for the production of ketocarotenoids in higher plants has the potential to lead to agriculturally useful products.
